# Intestinal perforation with systemic lupus erythematosus: A systematic review

**DOI:** 10.1097/MD.0000000000034415

**Published:** 2023-08-04

**Authors:** Ya Lan Chen, Jie Meng, Cong Li

**Affiliations:** a Department of Gastroenterology, The Affiliated Hospital of Hebei University, Baoding, P.R. China; b Department of Hepatobiliary Surgery, The Affiliated Hospital of Hebei University, Baoding, P.R. China.

**Keywords:** intestinal perforation, lupus mesenteric vasculitis, systemic lupus erythematosus

## Abstract

Intestinal perforation (IP) is a rare complication of systemic lupus erythematosus (SLE), and the timely diagnosis and treatment of IP are necessary to prevent death. In this study, the clinical features of IP in SLE were described in an attempt to enhance its understanding to reduce mortality. The clinical data of IP in SLE from 1984 to 2022 were retrospectively collected. A total of 18 patients were enrolled, and data on clinical symptoms, preoperative evaluation, surgical procedures, and postoperative outcomes were collected and retrospectively analyzed. The analysis included 15 females and 3 males, with a mean age of 49.2 years. Fifteen patients (83.3%) had a history of the disease for >5 years, and the SLE disease activity index score of 1 (5.6%) patient was <5 points and that of 17 (94.4%) patients was >10 points. A total of 9 (50%), 5 (27.7%), 3 (16.7%), and 1 (5.6%) patient had lesions in the rectum, colon, ileum, and both ileum and appendix, respectively. The cause of perforation in 12 (66.7%) patients was lupus mesenteric vasculitis and in 3 (16.7%) patients was chronic inflammation. Seven (38.9%) patients had other immune system diseases. All patients were treated with steroids and surgical treatment. However, 5 patients died after surgery. A disease duration of >5 years, SLE disease activity index score of >10, nonstandard use of steroids, and concomitant presence of other immune system diseases are the possible risk factors of IP in SLE. The most common site of perforation was the rectum, which was caused by lupus mesenteric vasculitis. The results suggest that the key to successfully manage such cases is early diagnosis, aggressive resuscitation, antibiotics, steroid therapy, and prompt surgical intervention.

## 1. Introduction

Systemic lupus erythematosus (SLE), an autoimmune disease, can affect and damage multiple organs and organ systems and mostly affects young women.^[1]^ While fever, joint pain, and rash are the most common symptoms of SLE, >50% of patients with SLE can also have gastrointestinal (GI) symptoms during the disease course.^[1]^ The most common GI symptoms include nausea, vomiting, and anorexia as well as abdominal pain, diarrhea, and abdominal distension. However, intestinal perforation (IP) is one of the most serious and rare complications of SLE, which may result in significant mortality. The early recognition of SLE-associated IP is critical in preventing misdiagnosis and treatment delay.

Until now, <20 cases of IP secondary to SLE have been reported in English and Chinese literature,^[2–16]^ and several issues with this complication have been left unresolved owing to the lack of large cohort studies. Thus, a scoping review of the literature published over the past 38 years on the topic of IP in SLE was performed to document the incidence, characteristics, clinical course, management and complications, and risk factors in an attempt to enhance its understanding to reduce mortality.

## 2. Materials and methods

As a systematic review, this study did not necessitate an institutional review board or research ethics committee approval.

### 2.1. Literature search

A literature search of the CBM Web, WanFang Data, Embase, Medline, WES, and PubMed (year range, 1984.1–2022.10) was performed to identify publications with “systemic lupus erythematosus and intestinal perforation” or “systemic lupus erythematosus and intestinal ulcer” or “systemic lupus erythematosus and intestinal necrosis” in the title or abstract without any language or time limits.

### 2.2. Eligibility criteria

**Figure 1.**
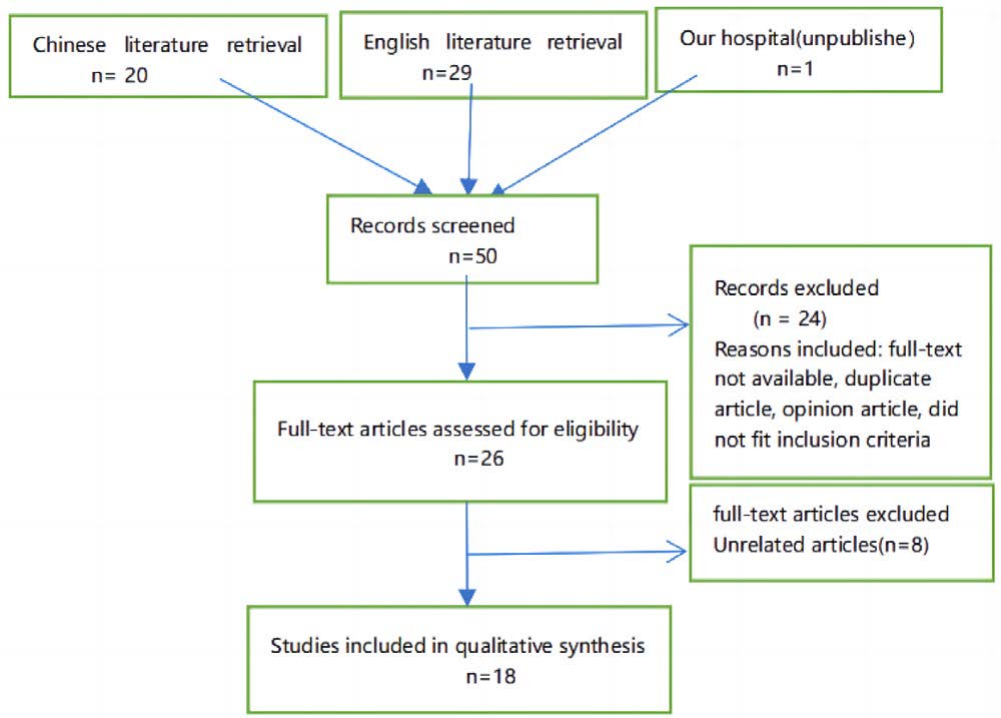
Literature search chart.

The articles included for analysis fulfilled the following primary criteria: SLE diagnosis was consistent with the definition given by the American College of Rheumatology,^[17]^ whereas acute abdomen was classified as per the International Classification of Diseases^[18]^ (ninth edition); the disease was diagnosed by rheumatologists and general surgeons and the SLE activity was evaluated using the SLE disease activity index (SLEDAI)^[19]^ standard; and an abdominal X-ray or computed tomography (CT) confirmed gas under the diaphragm, indicating bowel perforation. Confirmed duplicate cases, suspected duplicate cases, and cases with incomplete data were the exclusion criteria (Fig. 1). All identified cases with complete data were collected. A total of 17 cases^[2–16]^ reported in literature and 1 unpublished case from our department were included in the analysis. Thus, this retrospective study analyzed 18 patients (Table 1).

**Table 1:** Clinical features of patients with SLE-IP (N = 18).

No	Age	Sex	Disease	Activity	Diseased	Operation	Prognosis	Pathogeny	References
			Duration (yr)		Region	Method			
1	33	F	5	Y	Rectum	SRC	SS	LMV	^[2]^
2	46	F	5	Y	Rectum	SRC	SS	LMV	^[2]^
3	35	F	10	Y	Colon	SRC	Good	LMV	^[2]^
4	50	F	21	Y	Rectum	LRC	Good	CI	^[3]^
5	52	F	0	Y	Colon	LRC	SS	LMV	^[4]^
6	31	F	6	Y	Ileum	LRC	Die	LMV	^[5]^
7	48	F	5	Y	Rectum	SRC	Die	LMV	^[6]^
8	24	F	9	Y	Ileum	LRC	Die	LMV	^[7]^
9	64	F	21	Y	Ileum	LRC	Die	Lymphoma	^[8]^
10	22	F	0	Y	Ileum	LRC	Good	LMV	^[9]^
11	30	M	2	Y	Rectum	SRC	TLEV	LMV	^[10]^
12	32	M	10	Y	Rectum	LRC	Good	LMV	^[11]^
13	52	F	19	Y	Rectum	LRC	Good	LMV	^[12]^
14	41	M	25	N	Rectum	LRC	Good	CI	^[13]^
15	44	F	16	Y	Ileum+Appendix	LRC	SS	LMV	^[14]^
16	44	F	10	Y	Colon	LRC	Die	SP	^[15]^
17	58	F	19	Y	Colon	SRC	Good	CMV	^[16]^
18	54	F	15	Y	Rectum	SRC	Sinus tract	CI	Our hospital

CI = chronic inflammation, CMV = cytomegalovirus, F = female, IP = intestinal perforation, LMV = lupus mesenteric vascuitis, LRC = lesion resection + colostomy, M = male, M = methylprednisolone, N = no, SLE = systemic lupus erythematosus, SP = stercoral perforation, SRC = suture repair + colostomy, SS = septic shock, TLEV = thrombus of lower extremity veins, Y = yes.

### 2.3. Study selection

Data abstraction was independently performed by 2 investigators (YC and JM) using a standardized data collection form. Any discrepancies in data interpretation were resolved through formal discussion and re-review of the studies and by consulting another author (CL). If data from a single study were reported in more than 1 article, the duplicate cases were removed. However, if the same population group was analyzed in more than 1 publication, the studies were included if the study periods differed.

### 2.4. Clinical evaluation

The data from 18 cases of SLE-IP, included clinical characteristics as well as the pathological parameters, complications, treatment methods, and descriptive epidemiological outcomes.

For comparison with SLE-IP, 18 SLE patients with GI but without IP were randomly selected among those who were treated in our hospital.

### 2.5. Statistical analysis

Continuous data were tested for normal distribution using the Kolmogorov–Smirnov test. Normally distributed continuous data are presented as mean ± standard deviation and were analyzed using Student *t* test. Non-normally distributed data are presented as median (range) and were analyzed using the Mann–Whitney *U* test. Categorical data are presented as frequencies and were analyzed using the chi-squared test or Fisher’s exact test, as appropriate. Factors significantly different between the groups were entered into a logistic regression model to identify those associated independently with the development of IP. SPSS 22.0 software (SPSS Statistics for Windows, Version 22.0, Chicago, SPSS Inc) was used for statistical processing, and data are expressed as percentages. Two-tailed *P* values of <.05 were considered statistically significant.

## 3. Results

### 3.1. Demographic characteristics of the patients

Of the 18 patients, 15 were female and 3 were male, with a mean age of 49.2 years. Two (11.1%) patients were newly diagnosed with SLE, 1 had the disease for <5 years, and 15 (83.3%) had the disease for >5 years (Table 1). A total of 16 (88.8%) patients were treated with steroids, with doses between 5 and 10 mg/d, for a long time. Long-term immunosuppressant was prescribed to 8 (44.4%) patients, with four each receiving azathioprine and hydroxychloroquine, whereas 2 (11.1%) were not on any steroid therapy owing to undiagnosed SLE (Table 2).

**Table 2:** Clinical characteristics of patients with SLE and/without IP.

	SLE with IP (N = 18)	SLE without IP (N = 18)	*P*
Age, yr (mean ± SD)	42.22 ± 11.95	39.33 ± 3.7	.334
Duration, yr (mean ± SD)	11 ± 7.78	7 ± 1.2	.038*
Male	3 (16.7%)	1 (5.6%)	.603
Female	15 (83.3%)	17 (94.4%)	
Clinical manifestations of SLE with acute abdomen, n (%)			
Abdominal pain	18 (100%)	17 (94.4%)	1.000
High fever	13 (72.2%)	1 (5.6%)	<.001*
Nausea	11 (61.1%)	11 (61.1%)	1.000
Vomiting	11 (61.1%)	8 (44.4%)	.505
Bloody stools	3 (16.7%)	0	.229
Diarrhea	3 (16.7%)	5 (27.8%)	.691
SLEDAI score			
<4 points	1 (5.6%)	7 (38.9%)	.045*
>10 points	17 (94.4%)	11 (61.1%)	
Complications	7 (38.9%)	2 (11.1%)	.121
Rheumatoid arthritis	2 (11.1%)	1 (5.6%)	1.000
Ulcerative colitis	1 (5.6%)	0	1.000
Raynaud’s syndrome	1 (5.6%)	1 (5.6%)	1.000
Autoimmune anemia	1 (5.6%)	0	1.000
Vertical myelitis	1 (5.6%)	0	1.000
Chronic lymphocytic thyroiditis	1 (5.6%)	0	1.000
Pathogeny			.005*
LMV (%)	12 (66.7%)	5 (27.8%)	.044*
CI (%)	3 (16.7%)	13 (72.2%)	.002*
Prognosis			.010*
Serious complications	7 (38.9%)	2 (11.1%)	.121
Good	6 (33.3%)	15 (83.3%)	.006
Die	5 (27.8%)	1 (5.6%)	.177

Comparison between the 2 groups showed that the onset time of more than 10 years and SLEDAI score > 10 were risk factors for SLE with IP.

CI = chronic inflammation, IP = intestinal perforation, LMV = lupus mesenteric vascuitis, SLE = systemic lupus erythematosus, SLEDAI = SLE disease activity index.

* *P* < .05.

### 3.2. Comparison of SLE with IP and SLE with GI

Table 2 shows the demographic and clinical features of SLE-IP and SLE-GI cases. In the univariate analyses, the former were more likely to have a longer duration of illness and more pronounced GI symptoms (*P* < .05). IP occurred in the active phase of the disease, with the SLEDAI score of >10 points; however, GI symptoms occurred in remission. Approximately 38.9% of patients with SLE-IP were associated with multiple other immune system diseases, including rheumatoid arthritis (11.1%), ulcerative colitis (5.6%), Raynaud’s syndrome (5.6%), autoimmune anemia (5.6%), vertical myelitis (5.6%), and chronic lymphocytic thyroiditis (5.6%), which may be risk factors for IP (Table 2).

### 3.3. Radiographic images

Abdominal CT was performed in 15 (83.3%) patients: free gas under the diaphragm was detected in all 15 patients, whereas intestinal obstruction was detected in 2 (11.1%), abdominal effusion in 4 (22.2%), and intestinal wall thickening in 6 (33.3%). Abdominal X-ray performed for the remaining 3 patients indicated free gas in the abdominal cavity. Four (22.2%) patients also underwent colonoscopy, and intestinal ulcers were found in all of them (Table 3).

**Table 3:** Preoperative examination and treatment (n = 18).

Imaging examination	
CT	15 (83.3%)
Subphrenic free air	15 (83.3%)
Bowel wall thickening	6 (33.3%)
Intestinal obstruction	2 (11.1%)
Abdominal effusion	4 (22.2%)
X-ray	3 (16.7%)
Subphrenic free air	3 (16.7%)
Enteroscopy	
Intestinal ulcers	4 (22.2%)
Preoperative therapeutic intervention	
Glucocorticoid	16 (88.8%)
Prednisone acetate	12 (66.6%)
Methylprednisolone	4 (22.2%)
No	2 (11.1%)
Immunosuppressant	8 (44.4%)
Hydroxychloroquine	4 (22.2%%)
Azathioprine	4 (22.2%)
Antibiotics	18 (100%)

CT and X-ray can show the appearance of perforation, 88.8% patients had long-term use of glucocorticoids.

CT = computed tomography.

### 3.4. IP sites and pathological sections

Nine (50%) patients had lesions in the rectum, 4 (22.2%) had lesions in the colon, 4 (22.2%) had lesions in the ileum, and 1 (5.6%) had lesions both in the ileum and appendix.

**Figure 2.**
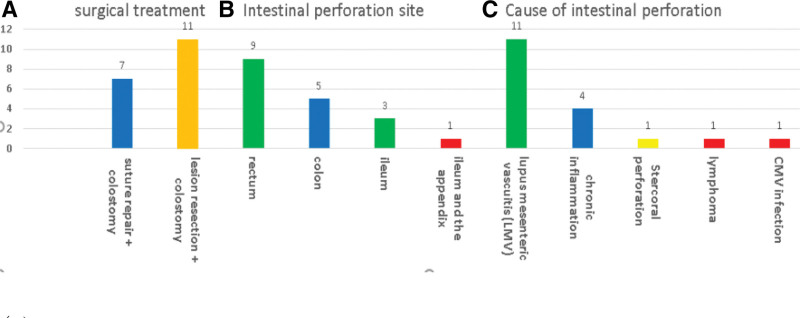
Location and cause of SLE-IP (n = 18). IP = intestinal perforation, SLE = systemic lupus erythematosus.

Perforation was caused by lupus mesenteric vasculitis (LMV), chronic inflammation, stercoral perforation, lymphoma, and cytomegalovirus infection in 12 (66.7%), 3 (16.7%), 1 (5.6%), and 1 (5.6%) patient, respectively (Fig. 2). Table 2 shows that LMV was the main cause of IP. (*P* < .05).

### 3.5. Treatment and prognosis

All patients were treated surgically: 7 (38.9%) underwent repair suturing with colostomy and 11 (61.1%) underwent lesion resection with colostomy. Patients were also treated with intravenous high-dose methylprednisolone (1 mg/kg/d) after surgery; the potent antibiotic sulperazon had a better anti-inflammatory effect in these patients (Table 3).

Four (22.2%) patients developed septic shock and were treated with anti-inflammatory medication and mechanical ventilation and 1 (5.6%) patient developed intestinal sinus, which remained unhealed after 3 years of follow-up. In addition, 1 (5.6%) patient developed lower extremity deep vein thrombosis, which improved after treatment; 5 (27.7%) patients died because of pulmonary embolism, lymphoma, multiple organ failure, re-perforation, and septic shock after surgery, respectively; and 7 (38.9%) patients recovered completely. Table 2 shows that compared with the SLE-GI group, the mortality of patients with SLE-IP was higher and the prognosis was worse (*P* < .05).

## 4. Discussion

SLE-mediated damage to the digestive system can manifest in the form of liver damage, intestinal pseudo-obstruction, protein-losing enteropathy, autoimmune pancreatitis, and other complications.^[20]^

Intestinal perforation at the onset of SLE is a rare and most serious complication, and it can be life threatening if not treated promptly.

The risk factors of acute abdomen should be identified as soon as possible when acute abdomen and SLE are present simultaneously. SLE mostly affects young women,^[20]^ and in our study, the female:male ratio was 5:1, with a mean age of 49.2 years, suggesting that the sex ratio of SLE complicated with IP is still biased toward female patients, albeit with a higher mean age. Acute abdomen usually appears several months to several years after the onset of SLE, but it may also appear as the first symptom. The study results revealed that 83.3% of the patients who developed IP had SLE for >5 years, indicating that a 5-year illness is a risk factor of IP. However, 2 patients with IP were newly diagnosed with SLE, which adds to the complexity of this complication in SLE. Regarding the SLEDAI score (Table 2), 1 patient had a score of <5, whereas 17 had a score of >10, suggesting that the disease is more active in SLE-associated IP. According to Medina et al,^[21]^ acute abdomen is extremely rare when the SLEDAI score is <5, which is consistent with the results of the present study. The results of 16 patients who were on steroids with doses between 5 and 10 mg/d and 7 patients who had other immune-related diseases indicated that nonstandard steroid therapy along with the presence of other immune diseases are also the risk factors of IP. Owing to the small sample size, the risk factors of IP among patients with SLE could not be fully delineated; however, Table 2 shows that females with higher baseline disease activity, longer disease duration, and nonstandard steroid use appear to be the major risk factors.

Patients with SLE may suddenly present with severe and persistent abdominal pain, accompanied by nausea and vomiting and even bloody stools. Thus, clinicians should be vigilant for IP as acute abdomen is more difficult to accurately and timely diagnose as the first manifestation of SLE. Both abdominal X-ray and CT can detect subdiaphragmatic gas to diagnose IP; however, in the present study, abdominal CT also revealed bowel wall edema, mesenteric abnormalities, ascites, and intestinal obstruction in SLE, suggesting that abdominal CT provides more important information for the detection and diagnosis of IP. Colonoscopy can detect ulcers in the intestines (Figure S1, Supplemental Digital Content, http://links.lww.com/MD/J344), but caution must be taken as colonoscopy may aggravate the perforation.

The most common pathological lesions in the GI tract of patients with SLE are caused by chronic, nonspecific mucosal inflammation as well as ischemic changes due to vascular lesions.^[22]^ Thus, the lack of blood supply to the intestinal area can lead to ulceration, infarction, and eventually perforation.^[23]^ In the present study, lupus mesenteric vascuitis was the main cause of perforation in 12 cases, which is consistent with the results reported in literature^[21]^; however, the presence of malignant disease, infection, nonspecific inflammation, stercoral perforation, and other complications may also lead to perforation. To the best of our knowledge, the most common sites of intestinal vascular involvement in patients with SLE are the jejunum and ileum. Owing to the abundant vasculature derived from the inferior mesenteric and internal iliac arteries, few cases of SLE-related rectal disease exist in literature.^[24]^ Figure 2 shows that most perforations were in the rectum, followed by the colon, and that the rectal perforation site was isolated, which is not in line with the existing literature.^[24]^ Several reasons for rectum ulceration have been proposed, including blood flow disturbances causing rectal ischemia^[25]^ as well as prolonged fecal mass retention in the rectal cavity and increased intestinal lumen pressure.

As IP in patients with SLE may be life threatening, early diagnosis and prompt treatment are critical. In the current study, all patients responded well to high-dose prednisolone (1 mg/kg/d), but 5 (27.7%) patients died because of pulmonary embolism, lymphoma, multiple organ failure, re-perforation, and infection shock after surgery, respectively. These results emphasize the importance of early laparotomy because of high mortality. In conclusion, the importance of early laparotomy cannot be emphasized enough, and prednisolone, potent antibiotics, and organ function support are important for treating symptoms, preventing complications, and improving prognosis.

Taken together, the present study revealed that IP in SLE is rare, the perforation site is mostly in the rectum, and perforation is mainly caused by LMV in the active stage of the disease. Surgery in combination with steroids and potent antibiotics is the key for treating IP in SLE. However, the study was limited by the small sample size as well as the single-center and retrospective study design, and accurate predictions about the associated risk factors and optimal treatment could not be made. Therefore, further studies on the clinical characteristics of IP in SLE should be more rigorous with a prospective cohort design and larger sample size or as a multicentric randomized controlled clinical trial. Our results provide cues to establish a better management strategy for IP in patients with SLE.

## Author contributions

**Investigation:** Jie Meng.

**Methodology:** Ya Lan Chen, Jie Meng.

**Software:** Ya Lan Chen, Cong Li.

**Validation:** Ya Lan Chen, Cong Li.

**Visualization:** Ya Lan Chen.

**Writing – original draft:** Ya Lan Chen.

**Writing – review & editing:** Cong Li.

## Supplementary Material


