# Supplementary figures and images for: Intestinal perforation with systemic lupus erythematosus: A systematic review
Source: Medicine (Baltimore). 2023 Aug 4;102(31):e34415. doi: 10.1097/MD.0000000000034415 (PMC10402941; doi:10.1097/MD.0000000000034415)

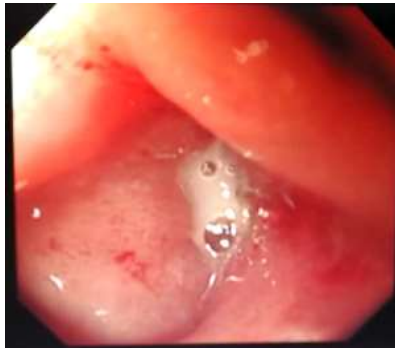

Supplementary Figure 1. The colonoscopy shows rectal ulceration with white pus covering

Supplement: Supplementary file 1 [file medi-102-e34415-s001.pdf]
